# Pathogenetic Interplay Between IL-6 and Tryptophan Metabolism in an Experimental Model of Obesity

**DOI:** 10.3389/fimmu.2021.713989

**Published:** 2021-07-30

**Authors:** Giada Mondanelli, Elisa Albini, Elena Orecchini, Maria Teresa Pallotta, Maria Laura Belladonna, Giovanni Ricci, Ursula Grohmann, Ciriana Orabona

**Affiliations:** ^1^Department of Medicine and Surgery, University of Perugia, Perugia, Italy; ^2^Service Center for Pre-clinical Research, University of Perugia, Perugia, Italy

**Keywords:** experimental obesity, tryptophan metabolism, indoleamine 2, 3 dioxygenase 1 (IDO1), tocilizumab (TCZ), white adipose tissue (WAT), IL-6 receptor (IL-6R), high fat diet (HFD)

## Abstract

Obesity is a metabolic disease characterized by a state of chronic, low-grade inflammation and dominated by pro-inflammatory cytokines such as IL-6. Indoleamine 2,3-dioxygenase 1 (IDO1) is an enzyme that catalyzes the first step in the kynurenine pathway by transforming l-tryptophan (Trp) into l-kynurenine (Kyn), a metabolite endowed with anti-inflammatory and immunoregulatory effects. In dendritic cells, IL-6 induces IDO1 proteasomal degradation and shuts down IDO1-mediated immunosuppressive effects. In tumor cells, IL-6 upregulates IDO1 expression and favors tumor immune escape mechanisms. To investigate the role of IDO1 and its possible relationship with IL-6 in obesity, we induced the disease by feeding mice with a high fat diet (HFD). Mice on a standard diet were used as control. Experimental obesity was associated with high IDO1 expression and Kyn levels in the stromal vascular fraction of visceral white adipose tissue (SVF WAT). IDO1-deficient mice on HFD gained less weight and were less insulin resistant as compared to wild type counterparts. Administration of tocilizumab (TCZ), an IL-6 receptor (IL-6R) antagonist, to mice on HFD significantly reduced weight gain, controlled adipose tissue hypertrophy, increased insulin sensitivity, and induced a better glucose tolerance. TCZ also induced a dramatic inhibition of IDO1 expression and Kyn production in the SVF WAT. Thus our data indicated that the IL-6/IDO1 axis may play a pathogenetic role in a chronic, low-grade inflammation condition, and, perhaps most importantly, IL-6R blockade may be considered a valid option for obesity treatment.

## Introduction

IL-6 is a pleiotropic cytokine that modulates a diverse array of functions relevant to hematopoiesis, tissue homeostasis, metabolism, and immunity (1). Its deregulation is associated with several diseases, including chronic inflammation, autoimmune disorders, and cancer. Inflammatory arthritis can indeed be successfully treated with tocilizumab (TCZ), a monoclonal antibody capable of binding and blocking the IL-6R subunit of the IL-6 receptor (2). In cancer, IL-6 drives proliferation, survival, invasiveness, and metastasis of tumor cells, while strongly suppressing the anti-tumor immune response (3).

Indoleamine 2,3-dioxygenase 1 (IDO1) is an enzyme that catalyzes the first, rate-limiting step in the kynurenine pathway, leading to depletion of the essential amino acid l-tryptophan (Trp) and production of a series of immunoregulatory molecules collectively known as kynurenines (4, 5). Both effects – namely, Trp starvation and kynurenine (Kyn) production – are involved in the generation of regulatory T cells (6). Highest IDO1 expression is detectable in dendritic cells (DCs), especially in the presence of IFN-γ (4). In contrast, the presence of a microenvironment dominated by IL-6 favors IDO1 targeting for proteasomal degradation *via* recruitment of the E3 ubiquitin ligase complex (7). Therefore, in contrast to IFN-γ, IL-6 reduces IDO1 half-life, thus interrupting immunosuppressive mechanisms and favoring a pro-inflammatory phenotype in the DCs. However, in human cancer [in which IDO1 is often overexpressed (8)], IL-6 sustains constitutive IDO1 expression (9). Moreover, inhibition of IL-6 production by tumor cells reduces IDO1 expression and tumor-mediated immunosuppressive effects (9).

Obesity is a metabolic disorder characterized by a chronic, low-grade inflammatory state and associated with the development of numerous comorbid conditions, including insulin resistance and type 2 diabetes (10). The inflammatory program is activated early in adipose expansion and during chronic obesity, permanently skewing the immune system to a pro-inflammatory phenotype characterized by M1 macrophages and the production of IL-1β, IL-6, IFN-γ, and TNF-α (11). Interestingly, the chronic, low-grade inflammation associated to obesity also promotes the development of numerous tumors, such as liver and colorectal cancer (12). Unexpectedly, in a previous study, mice fed with a high fat diet (HFD) and lacking IDO1 expression gained less weight, had a lower fat mass and better glucose tolerance (13). Depletion of IDO1 was found to increase the production of protective Trp metabolites by gut bacteria. Consistent with the observation in mice, obese patients have lower Trp and higher Kyn in plasma (14).

In the present study, we investigated the possible relationship between IL-6 and IDO1 in obesity. To do so, we resorted to HFD-fed mice and found that (i) IDO1 and Kyn production increase in the stromal vascular fraction of visceral white adipose tissue (SVF WAT) along weight gain, increased fat mass, and reduced glucose tolerance and insulin sensitivity; (ii) administration of TCZ abrogates IDO1 expression and Kyn production in SVF WAT, greatly reduces weight gain and adipose tissue hypertrophy, increases insulin sensitivity, and induces a better glucose tolerance. Therefore, our data indicated the existence of an aberrant interplay between IL-6 and IDO1 in obesity and the possibility to use IL-6R blockers for therapeutic purposes in obese patients.

## Materials and Methods

### Mice and *In Vivo* Treatments

Six- to eight-week-old male C57BL/6 mice were obtained from Charles River Breeding Laboratories and used for pharmacological studies. *Ido1^−/−^* C57BL/6 mice were obtained from an internal breeding at the Plaisant S.r.l. animal facility. All animal studies were approved by the Italian Ministry of Health. Mice were fed with either a standard diet (SD) (Mucedola Srl) or high fat diet (HFD) containing 42% fat (Mucedola Srl). HFD was started at 8 weeks of age and continued for 10 wk or less with *ad libitum* access to water and food. Daily food intake was determined at 8 a.m. by weighing the metal cage top, including the food. The average WAT weight per mouse was determined by the ratio of the total weight of the visceral WAT isolated from mice to the number of mice analyzed in each experimental group. Six to eight mice were used in each treatment or control group. Impairment of glucose homeostasis was investigated by intraperitoneal (i.p.) glucose tolerance testing (IPGTT) at specific time points of HFD feeding. Briefly, 16−h fasted mice were administered i.p. with 1 g/kg d-glucose. Blood glucose concentrations were measured before anesthesia by tail incision using a digital glucometer (Roche). TCZ (Chugai Pharmaceutical Co.) or saline was administered i.p. at the dose of 5 mg/kg (15) every other day for 4 wk, in parallel with the diet-induced feeding, or twice a week for 6 wk, when the drug treatment was delayed 2 wk later the starting of HFD diet. Animals were sacrificed after anesthesia by i.p. administration of Avertin (125 mg/kg) for *ex vivo* analyses.

### Isolation of SVF and Morphometry of Adipose Tissues

Visceral white adipose tissue (WAT) was excised from mice and processed for SVF cell isolation as described (16). Briefly, tissues were cut into small pieces and digested in 1 mg/ml collagenase P (Roche) in HBSS for 40 min at 37°C. The digested tissues were passed through a 100-μm cell strainer to remove debris. After centrifugation, the floating cell layer and supernatant were removed and the cell pellet was washed with HBSS. Primary SVF cells were maintained in DMEM plus 10% FCS. For histology, 3–4 μm of paraffin-embedded sections of WAT were stained with hematoxylin and eosin and analyzed by light microscopy. For quantification of adipocyte size, sections were analyzed by a DM2500 Leica microscope equipped with Leica DFC420C digital camera (Leica microsystem). Adipocyte diameters were measured in 30 adipocytes per section (five sections for each WAT sample), and data analysis was performed using Leica Application Suite (LAS v3.8, Leica microsystems) for digital image processing.

### Determination of Insulin Sensitivity in Primary Hepatocytes

Insulin sensitivity was evaluated in primary hepatocytes isolated from mice euthanized at the end of the experiment. Specifically, the liver was cut into small pieces and perfused with a digestion medium containing 0.8 mg/mL of collagenase type IV (Sigma-Aldrich) in HBSS for 40 min at 37°C. Hepatocytes were dispersed in the medium using a pipette and filtered through a 100-μm cell strainer. After centrifugation, cells were washed with HBSS and kept in a serum-free medium for 1 h at 37°C before insulin stimulation. Hepatocytes were treated with 100 nM of insulin (Sigma-Aldrich) and incubated at 37°C for 5, 15, 30, and 60 minutes. Cells were then washed with ice-cold PBS and lysed with ice-cold RIPA buffer (50 mM Tris-HCl pH 7.4, 150 mM NaCl, 1% Nonidet P-40, 0.25% Na-deoxycholate) supplemented with Halt Protease inhibitor and Halt Phosphatase Inhibitor Cocktail (Thermo Scientific™). Cell lysates were immediately analyzed by immunoblot.

### Western Blot Analyses

These procedures were done as described (17–19). Briefly, protein lysates were subjected to SDS-PAGE and electro-blotted onto 0.2 μm nitrocellulose membranes (Bio-Rad). Membranes were blocked with 5% non-fat dried milk in TBS and probed with a primary antibody specific for the protein of interest in combination with an appropriate horseradish peroxidase-conjugated antibody (Millipore), followed by enhanced chemiluminescence (ECL) (Bio-Rad). IDO1 was investigated with a rabbit monoclonal anti-mouse IDO1 antibody (cv152) (20) in SVF WAT cells. Akt and its phosphorylated form were revealed by specific anti-Akt and -pAkt (Ser 473) antibodies (Cell Signaling) in primary hepathocytes. Anti-β-tubulin (Sigma-Aldrich) was used as a normalizer.

### Kynurenine and Cytokine Determinations

IDO1 activity was measured in terms of the ability to metabolize Trp to Kyn. Briefly, SVF WAT cells, at the concentration of 1.5 x 10^6^ cells/ml, were mantained in DMEM plus 10% FCS at 37°C in a humidified 7% CO_2_ incubator. Kyn concentration in the culture supernatants was measured by high performance liquid chromatography after 24 h of incubation (21, 22). Mouse cytokines (IL-1β, IL-4, IL-6, IL-10, IL-17A, IFN-γ, TGF-β, and TNF-α) were measured in 24−h SVF WAT culture supernatants by ELISA using specific kits (eBioscience and Thermo Fisher Scientific) and according to the manufacturer’s recommendations.

### Real-Time PCR

Real-Time PCR (for mouse *Ido1*, *Ucp1*, and *Gapdh*) analyses were carried out as described (17–19). Briefly, total RNA was extracted from SVF cells by TRIzol (Invitrogen) and reverse transcribed to cDNA with QuantiTect Reverse Transcription Kit (Qiagen). Real-time PCR was performed using SYBR Green detection and the following specific primers were used: *Ido1*, 5’- GATGTTCGAAAGGTGCTGC-3’ and 5’-GCAGGAGAAGCTGCGATTTC-3’; *Ucp1*, 5’-TCAGGATTGGCCTCTACGAC-3’ and 5’-TGCCACACCTCCAGTCATTA-3’; *Gapdh*, 5’-CTGCCCAGAACATCATCCCT-3’ and 5’-ACT TGG CAG GTT TCT CCA GG-3’. Values (means ± SD of triplicate determination) were expressed as the ratio of *Gapdh*-normalized transcript expression in SVF cells from HFD-fed mice to *Gapdh*-normalized transcript expression in SVF cells from SD-fed mice (calibrator, in which the fold change = 1; dotted line).

### Statistical Analyses

Data are expressed as means, and error bars indicate standard deviation. At least three biological replicates were used for each measurement. The exact number of biological replicates for a specific experiment is indicated in the figure legends. A ‘‘biological replicate’’ is a mouse for *in vivo* studies. A single value for a biological replicate could be the average of values from technical replicates of the same biological replicate, but statistical comparisons were made for averages of values from biological replicates. All statistical analyses were performed using Prism version 6.0 (GraphPad Software). Data were analyzed by two-tailed unpaired Student’s *t* test or 2-way ANOVA followed by *post hoc* Bonferroni’s test, when three or more samples were under comparison, respectively. Differences were considered significant with *p* < 0.05. Data are representative of two-three independent experiments.

## Results

**Figure 1:**
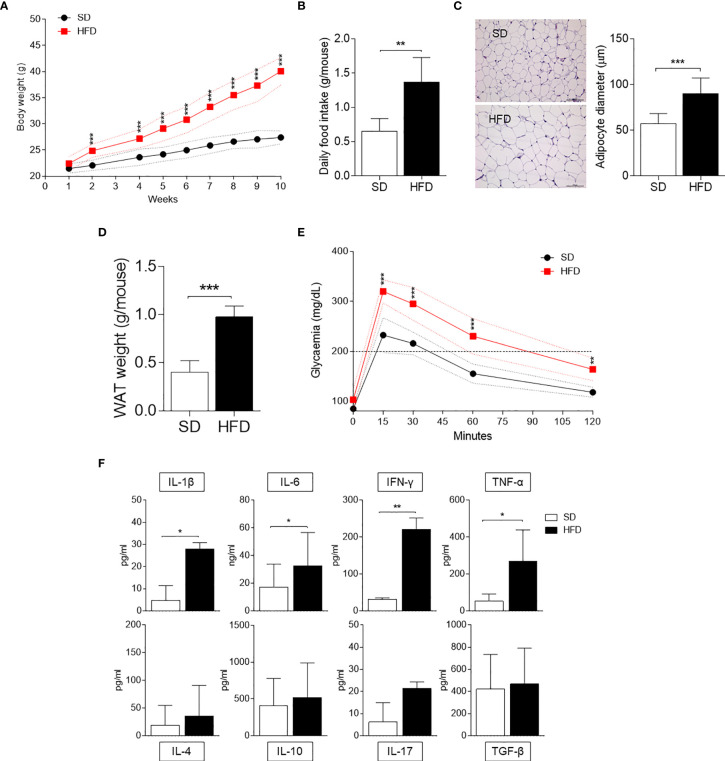
Obesity and inflammatory parameters of HFD-fed mice. **(A)** Body weight (g) of 6-wk male mice fed with high-fat diet (HFD, *n* = 10) for 10 wk compared with gender- and age-matched controls fed with a standard diet (SD, *n* = 10). **(B)** Average food intake (g) per mouse per day (*n* = 10, from two independent experiments). **(C)** Hematoxylin and eosin staining of visceral WATs (left panel, scale bars of 100 μm.). Analysis of adipocyte diameter (right panel). **(D)** Average WAT weight (g) per mouse (*n* = 5, from two independent experiments). **(E)** Intraperitoneal glucose tolerance test (IPGTT) after 10 weeks of HFD (*n* = 5, from two independent experiments). Glycaemia (mg/dl) was measured at different time points (0, 15, 30, 60, and 120 min) from the administration of glucose. **(F)** Levels of cytokines secreted by SVF WAT cells in 24-h culture supernatants. Results are represented as means ± S.D (*n* = 3 biological replicates, from two independent experiments). *p < 0.05, **p < 0.01, ***p < 0.001 HFD *versus* SD, two-tailed unpaired Student’s *t* test and multiple Student’s *t* test per row, corrected by *post hoc* Sidak-Bonferroni’s method.

We first examined HFD-fed mice in our setting in terms of several parameters typical of obesity, such as weight gain, daily food intake, and glucose tolerance. Mice fed with SD were used as control. We focused the analysis on WAT in terms of adipocyte hypertrophy and weight. Moreover, we measured the production of cytokines by SVF WAT cells [mainly containing macrophages, hematopoietic progenitor cells (21), and adipocyte precursor cells (22)]. Starting from 2 wk of feeding, mice on HFD showed significantly higher weights, which further increased over time reaching a gain of approximately 18 g in 10 wk (**Figure 1A**). At 10 wk of feeding, obese mice were characterized by a significant higher daily food intake (**Figure 1B**), WAT adipocyte diameter (**Figure 1C**), and weight (**Figure 1D**). Moreover, at the same time, obese mice exhibited higher blood glucose concentrations when challenged with the glucose tolerance test (**Figure 1E**). The cytokine profile of SVF WAT cells revealed a significantly higher release of pro-inflammatory IL-1β, IL-6, IFN-γ, and TNF-α but not of IL-4, IL-10, IL-17A, and TGF-β in HFD-fed mice (**Figure 1F**).

**Figure 2:**
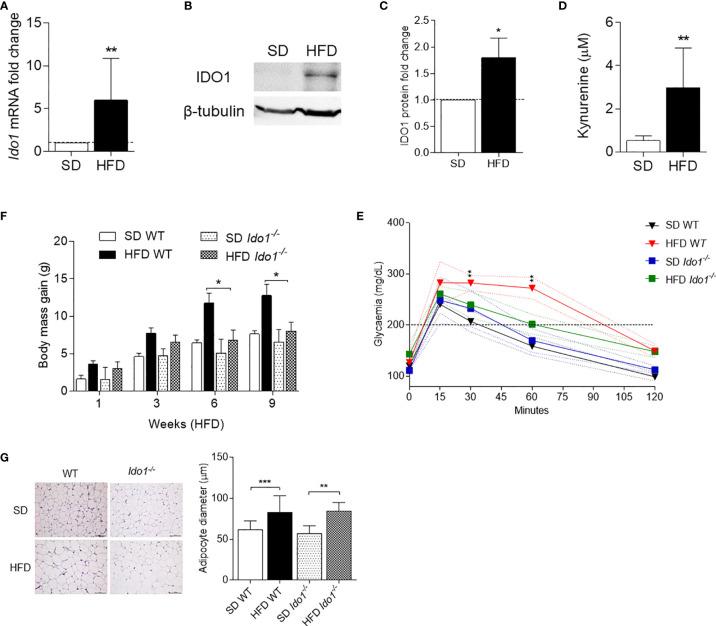
IDO1 expression and activity in diet-induced obesity. Expression of IDO1 gene **(A)** and protein **(B)** in SVF WAT cells of HFD *versus* SD mice after 10 weeks of diet. **(C)** Quantitative analysis of immunoblots from two independent *ex vivo* experiments, one of which represented in **(B)**. Data (mean ± S.D., *n* = 3 biological replicates) represent the ratio of tubulin-normalized IDO1 protein in SVF WAT from mice on HFD to that expressed in SD control counterparts. **(D)** Levels of Kyn (mean ± S.D., *n* = 3 biological replicates) secreted by SVF WAT cells in 24-h culture supernatants. *p < 0.05, **p < 0.01, HFD *versus* SD (two-tail unpaired Student’s *t* test for **C**, **D**). **(E)** Body weight gain of WT and *Ido1^−/−^* mice throughout 9 wk of high-fat diet (HFD, *n* = 10) treatment compared with gender- and age-matched controls fed with a standard diet (SD, *n* = 10). *p < 0.05, HFD WT *versus* HFD *Ido1^−/−^* mice, ANOVA followed by *post hoc* Bonferroni’s method. **(F)** Intraperitoneal glucose tolerance test (IPGTT) after 9 wk of HFD (*n* = 5, from two independent experiments). Glycaemia (mg/dl) at different time points (0, 15, 30, 60, and 120 min) from the administration of glucose. **(G)** Hematoxylin and eosin staining of visceral WAT (left panel, scale bars are 100 μm.). Analysis of adipocyte diameter (right panel). **p < 0.01, ***p < 0.001 HFD *versus* SD mice per genotype (ANOVA followed by *post hoc* Bonferroni’s method for **F, G**).

In order to evaluate IDO1 expression and activity in our setting, levels of IDO1 transcript and protein as well as release of Kyn, the main IDO1 product, were evaluated in SVF WAT cells. Results showed that, at 10 wk of feeding, obese mice expressed a 6-fold increase in *Ido1*-encoding transcripts (**Figure 2A**) and 2-fold in IDO1 protein expression (**Figures 2B, C**). Kyn release also increased 3-fold in the same SVF WAT cells (**Figure 2D**). We next compared the obesity parameters in wild-type (WT) and *Ido1^−/−^* mice, both fed with HFD. In agreement with previous data (13), results showed that IDO1-deficient mice gain significantly less weight (**Figure 2E**), and have a better glucose tolerance (**Figure 2F**), but a reduced adipocyte hypertrophy could not be observed (**Figure 2G**).

**Figure 3:**
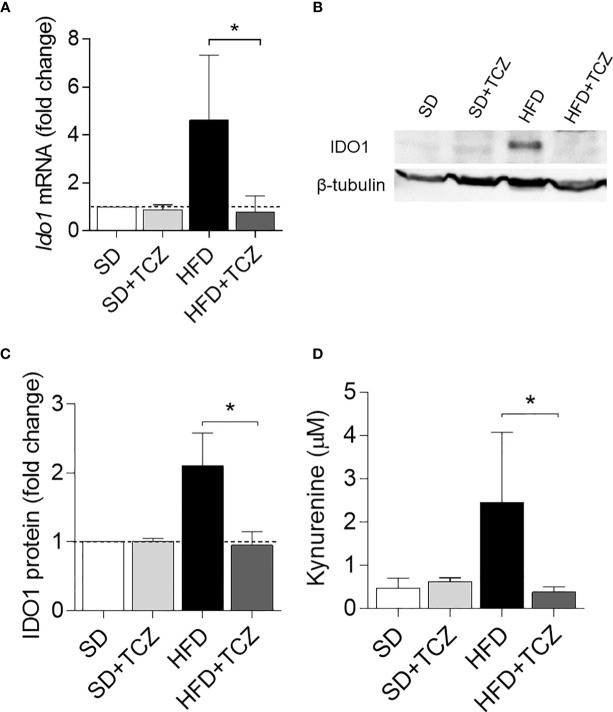
TCZ inhibits IDO1 expression in SVF WAT. **(A)** Gene transcription of *Ido1 in* SVF WAT cells after 9 wk of diet. Data (mean ± S.D., n = 3 biological replicates, from two independent experiments) represent the fold change expression of *Gapdh*-normalized transcripts in which the calibrator is represented by SVF WAT from SD-fed mice (fold change = 1; dotted line). **(B)** IDO1 protein expression in SVF WAT cells and quantitative analysis **(C)** of immunoblots from two independent *ex vivo* experiments, one of which shown in **(B)**. Data (mean ± S.D., n = 3 biological replicates, from two independent experiments) represent the ratio of tubulin-normalized IDO1 protein expression in SVF cells from HFD-fed mice to that expressed in SVF from animals on SD (n=3 mice per group). **(D)** Levels of Kyn (mean ± S.D., n = 3 biological replicates, from two independent experiments) secreted by SVF WAT cells in 24−h culture supernatants. *p < 0.05 (ANOVA followed by *post hoc* Bonferroni’s method for **A, C, D**).

**Figure 4:**
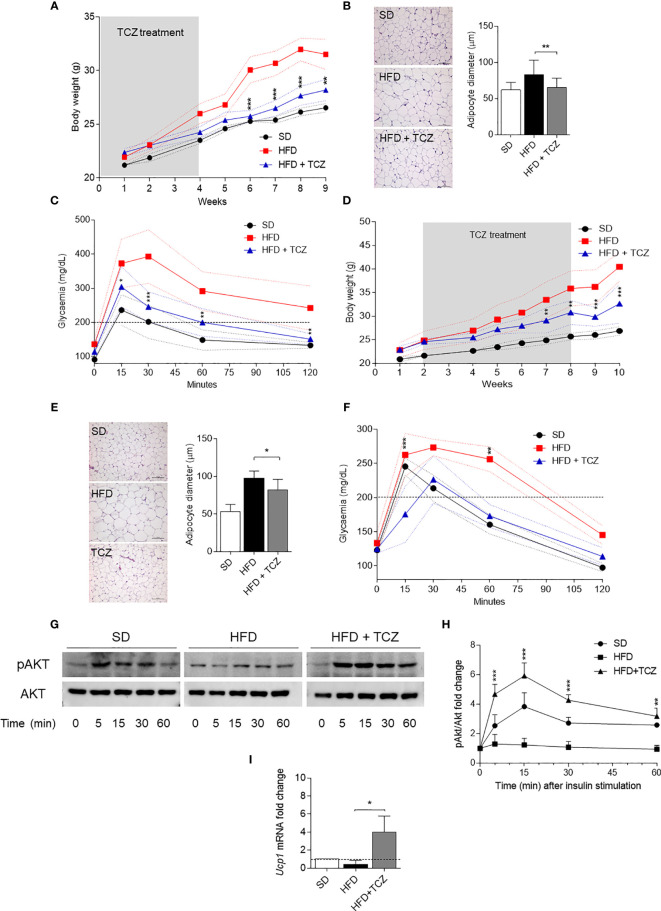
TCZ effects in diet-induced obesity. **(A, D)** Body weight (g) of HFD-fed mice receiving TCZ 5 mg/Kg (HFD TCZ, *n* = 8) or saline (HFD, *n* = 8) administered i.p. compared with gender- and age-matched controls fed with a standard diet (SD, *n* = 8). TCZ treatment started with HFD **(A)** or 2 wk later **(D)** and ended after 4 and 6 weeks, respectively, in A and D (grey box). **(B, E)** Hematoxylin and eosin staining of visceral WAT (left panel, scale bars of 100 μm.). Analysis of adipocyte diameter (right panel). **(C, F)** Intraperitoneal glucose tolerance test (IPGTT) at the end of TCZ treatment. Glycaemia (mg/dl) was measured at different time points (0, 15, 30, 60, and 120 min) from the administration of glucose. **(G, H)** Immunoblot and quantitative analysis of insulin-driven AKT phosphorylation in *ex vivo* hepatocytes from mice represented in **(D)**. Data from two independent experiments (means ± S.D., n = 3 biological replicates per group) represent the fold change of the pAKT/AKT ratio in hepatocytes stimulated with insulin at the indicated times in which the calibrator is represented by pAKT/AKT ratio at time 0. **(I)** Gene transcription of *Ucp1 in* SVF WAT cells from mice represented in **(D)**. Data (mean ± S.D., n = 3 biological replicates per group) represent the fold change expression of *Gapdh*-normalized transcripts in which the calibrator is represented by samples from SVF WAT from SD-fed mice (fold change=1; dotted line). *p < 0.05, **p < 0.01, ***p < 0.001; HFD TCZ *versus* HFD (ANOVA followed by *post hoc* Bonferroni’s method).

Because IL-6 is a cytokine widely recognized to play a major role in obesity and is also known to exert dichotomic effects on IDO1 expression (7, 13), we investigated the possible effect of the cytokine on IDO1 expression and activity in the WAT of diet-induced obese mice. To do so, we resorted to TCZ, a monoclonal antibody blocking the activation of the IL-6 receptor already used by us in nonobese diabetic mice (15). More specifically, WT mice on HFD were administered i.p. with TCZ at the dose of 5 mg/kg every other day for 4 wk (15). Saline injection and TCZ treatment of SD fed mice were used as controls. We observed that TCZ treatment completely abrogated IDO1 expression in terms of transcripts (**Figure 3A**), protein (**Figures 3B, C**), and Kyn release (**Figure 3D**) in SVF WAT cells of HFD-fed mice at the end of the feeding. No IDO1 modulation was observed in the SVF WAT cells of the TCZ-treated mice on SD, thus suggesting a dominant role of IL-6 in upregulating IDO1 in the adipose tissue of obese animals. Perhaps most impressively, TCZ administration rendered the effects of HFD similar to those of a standard diet. Indeed, no weight gain (**Figure 4A**) and adipocyte hypertrophy (**Figure 4B**) could be observed in TCZ-treated obese mice as compared to untreated obese mice. Likewise, glucose tolerance of TCZ-treated mice on HFD was indistinguishable from that of mice on standard diet (**Figure 4C**). TCZ effects could also be observed when the drug administration was delayed at 2 wk of feeding with HFD, when obese mice had already gained weight (**Figures 4D–F**). To confirm the glucose tolerance induced by TCZ treatment as a surrogate marker of insulin responsiveness, we also evaluated the insulin-induced AKT phosphorylation (pAKT) (23) in primary hepatocytes from the experimental groups shown in **Figure 4D**. In contrast to control mice, very low levels of pAKT could be induced in the cells from HFD-fed mice. However, the TCZ treatment completely restored insulin sensitivity by significantly increasing the ratio pAKT/AKT (**Figures 4G, H**). In order to see whether TCZ could also have an impact on browning, i.e., the process by which some adipocytes within WAT acquire properties of brown adipocytes (“beiging” effect), the transcript expression of uncoupling protein-1 [UCP-1; i.e., provoking energy dissipation by uncoupling respiration from ATP synthesis (24)] was evaluated. Results showed that the *Ucp1* gene expression was significantly upregulated by TCZ treatment in WAT of HFD-mice as compared to untreated animals (**Figure 4I**). Differently from HFD-fed mice, both insulin-induced phosphorylation of AKT in primary hepatocytes and induction of the *Ucp1* gene resulted to be insensitive to TCZ treatment in mice fed with SD (**Supplementary Figure 1**).

## Discussion

Low-grade, chronic inflammation has also been termed metaflammation, i.e., an inflammatory state orchestrated by metabolic cells in response to excess nutrients and energy (25). In metabolic organs including the liver, pancreas, and adipose tissue, the interaction of metabolic cells with the stromal components represents an important determinant in the maintenance of tissue homeostasis, thus preventing metaflammation.

Apart from its function as an energy storage, WAT is a large metabolically and immunologically active endocrine organ composed of mature adipocytes in addition to adipose-derived stem cells, fibroblasts, endothelial cells, and a wide range of immune cells (i.e., mainly macrophages) that overall constitute the SVF WAT (26). Depending on the microenvironmental conditions, adipose-derived stem cells can differentiate into either white or brown-like adipocyte phenotypes (27). When caloric intake exceeds caloric expenditure, WAT becomes hypertrophied and heavily infiltrated by immune cells with a pro-inflammatory phenotype, causing metaflammation and obesity often associated with insulin resistance.

In animal models, it is well documented that HFD induces metaflammation (25), with the production of pro-inflammatory cytokines such as TNF-α, IL-1β, and IL-6 by the adipose tissue (28). By using HFD-fed mice as an experimental model of obesity, we indeed found increased levels of those cytokines as well as of IFN-γ in the culture supernatants of SVF WAT cells from obese animals as compared to their counterparts on SD. In the same cells, such pro-inflammatory profile was accompanied by high expression and activity of IDO1, an immunometabolic enzyme involved in Trp metabolism and endowed with potent anti-inflammatory and immunoregulatory properties when expressed in DCs (5, 29). As hypothesized previously (13), high IDO1 expression in WAT of obese mice could be caused by higher local levels of IFN-γ, the potent inducer of the enzyme (30). Lack of IDO1 expression ameliorated the disease in terms of weight gain and glucose tolerance but not of adipocyte hypertrophy, suggesting that Trp metabolism exerts pathogenetic rather than protective effects in obesity. Mitigating effects of IDO1 depletion have been ascribed to a rewiring of host to microbiota Trp metabolism producing a protective indole derivative and not to the absence of Kyn (13), the IDO1 product known to promote arterial vessel relaxation and thus pro-inflammatory effects (31). Therefore, our data would sustain the importance of the microbiota Trp metabolism in obesity.

In addition to IFN-γ, IDO1 expression can also be upregulated in macrophages by combinations of TNF-α, IL-1β, and IL-6 but not by the single cytokines (32). However, in human tumor cells, IL-6 alone can significantly upregulate the enzyme expression (9). Because remarkable similarities between adipose expansion and growth of solid tumors have been observed (22), we evaluated the *in vivo* IL-6 dependency of IDO1 expression and activity in obesity. Administration of TCZ, an IL-6R blocker, to HFD-fed mice brought the levels of IDO1 transcript and protein expressions as well as Kyn production to those of control animals, thus suggesting a major role of IL-6 rather than IFN-γ in upregulating Trp metabolism in the obese adipose tissue. Perhaps most importantly, the TCZ treatment, either commenced at 0 or 2 wk of HFD, profoundly changed all the parameters examined by us for obesity so far, such that HFD-mice were indistinguishable from their counterparts on SD. Of note, the monoclonal antibody significantly increased the expression of *Ucp1*, suggesting a beiging effect on the adipose-derived stem cell component of SVF WAT of obese animals that may greatly contribute to the overall therapeutic effect of TCZ. Because the TCZ treatment but not IDO1 depletion also reduced adipocyte hypertrophy, our data suggested that the pathogenic role of IL-6 in the disease goes beyond IDO1 and other IL-6−driven mechanisms may be at work.

The incidence of obesity and its serious complications, particularly cardiovascular and metabolic diseases, is steadily increasing worldwide. Unfortunately, no truly effective and safe therapeutic options are available yet. Targeting specific molecules of metaflammation with biologic drugs in the adipose tissue may provide novel opportunities of drug treatment. However, blockade of either IL-1β (33) or TNF-α (34) has shown limited success in obese patients. Besides a few number of studies in patients with rheumatoid arthritis aimed at evaluating the effects of obesity on drug effectiveness (35, 36), no clinical trial has been performed with TCZ in obese patients yet. In addition to provide the evidence for the existence of a pathogenetic IL-6/IDO1 axis in obesity, our data suggested that IL-6 blockade by TCZ may represent a promising therapeutic option for obese patients.

## Data Availability Statement

The raw data supporting the conclusions of this article will be made available by the authors, without undue reservation.

## Ethics Statement

The animal study was reviewed and approved by Italian Ministry of Health.

## Author Contributions

CO designed and supervised the study as a whole. GM and EA performed the majority of experiments. EO performed Kyn determinations. GR performed histological analyses. MB and MP helped with some experiments and provided reagents. UG and CO wrote the manuscript. All authors contributed to the article and approved the submitted version.

## Funding

This work was supported by Associazione Italiana per la Ricerca sul Cancro (AIRC 2019-23084; to UG) and the Italian Ministry of Education, University, and Research (PRIN2017- 2017BA9LM5 to CO).

## Conflict of Interest

The authors declare that the research was conducted in the absence of any commercial or financial relationships that could be construed as a potential conflict of interest.

## Publisher’s Note

All claims expressed in this article are solely those of the authors and do not necessarily represent those of their affiliated organizations, or those of the publisher, the editors and the reviewers. Any product that may be evaluated in this article, or claim that may be made by its manufacturer, is not guaranteed or endorsed by the publisher.
