# Supplementary figures and images for: Pathogenetic Interplay Between IL-6 and Tryptophan Metabolism in an Experimental Model of Obesity
Source: Front Immunol. 2021 Jul 30;12:713989. doi: 10.3389/fimmu.2021.713989 (PMC8361489; doi:10.3389/fimmu.2021.713989)

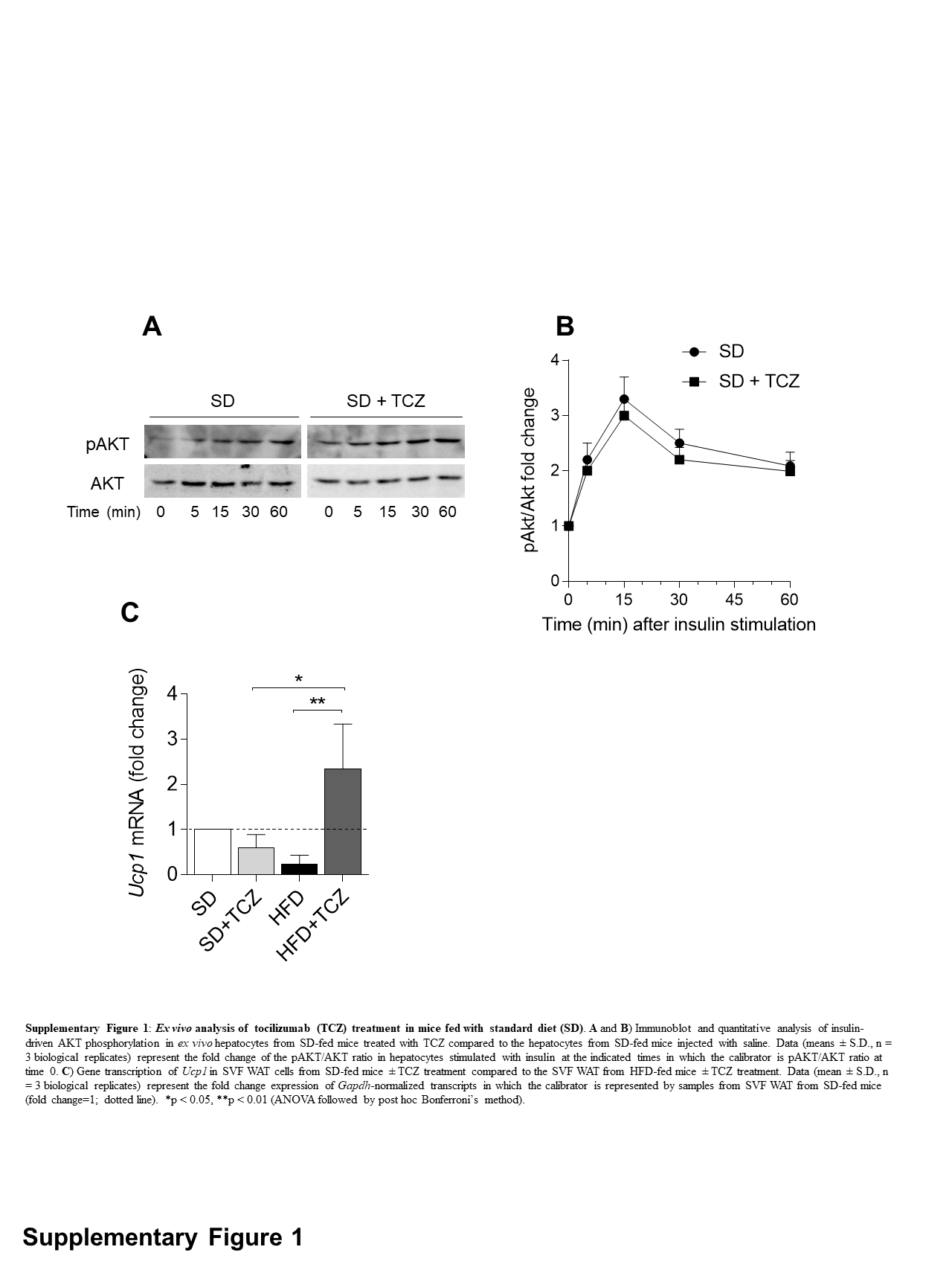

Supplement: Supplementary file 1 [file Image_1.tif]
